# Comprehensive analysis of somatic copy number alterations in clear cell renal cell carcinoma

**DOI:** 10.1002/mc.23164

**Published:** 2020-02-10

**Authors:** Takashi Tsuyukubo, Kazuyuki Ishida, Mitsumasa Osakabe, Ei Shiomi, Renpei Kato, Ryo Takata, Wataru Obara, Tamotsu Sugai

**Affiliations:** ^1^ Department of Molecular Diagnostic Pathology, School of Medicine Iwate Medical University Morioka Japan; ^2^ Department of Urology, School of Medicine Iwate Medical University Morioka Japan

**Keywords:** clear cell renal cell carcinoma, cluster analysis, patient prognosis, renal carcinogenesis, somatic copy number alteration

## Abstract

Somatic copy number alterations (SCNAs) are important biological characteristics that can identify genome‐wide alterations in renal cell carcinoma (RCC). Recent studies have shown that SCNAs have potential value for determining the prognosis of RCC. We examined SCNAs using the Affymetrix platform to analyze samples from 59 patients with clear cell RCCs (ccRCCs) including first cohort (30 cases) and second cohort (validation cohort, 29 cases). We stratified SCNAs in the ccRCCs using a hierarchical cluster analysis based on SCNA types, including gain, loss of heterozygosity (LOH), copy neutral LOH, mosaic, and mixed types. In this way, the examined two cohorts were categorized into two subgroups (1 and 2). Although the frequency of mixed type was higher in subgroup 1 than in subgroup 2 in the two cohorts, the association did not reach statistical significance. There was a significant difference in the frequency of metachronous metastasis between subgroups 1 and 2 (subgroup 2 > 1). In addition, subgroup 2 was retained in multivariate analysis of both cohorts. We examined whether there were specific alleles differing between subgroups 1 and 2 in both cohorts. We found that there was indeed a statistically significant difference in the 3p mixed types. Among the 3p mixed type, we found that 3p24.3 mixed type was inversely correlated with the presence of metachronous metastasis in ccRCC. The association was also retained in multivariate analysis in second cohort. We suggest that the 3p24.3 mixed type may be a novel marker to predict a favorable prognosis in ccRCC.

## 1 INTRODUCTION

Renal cell carcinoma (RCC) is the most common type of urological cancer, accounting for 2~3% of adult malignancies worldwide.^1^ Although RCC is a potentially curable disease, approximately 40% of patients with RCC die due to disease progression.^2^ RCC is characterized by heterogeneous histological features.^3^ The histological classification of RCC is important for histopathologists as well as urologists because such classification may help to determine patient treatment and prognosis of the disease.^3^ However, histological classification is limited in its ability to predict patient outcome, even in clear cell RCC (ccRCC).^4^, ^5^ In that regard, biological markers that predict patient outcome in ccRCC are available.^6^, ^7^, ^8^ Recently, somatic copy number alterations (SCNAs) have been used to predict tumor aggressiveness in ccRCC.^9^, ^10^ Several studies have demonstrated that the presence of multiple SCNAs is associated with overall patient survival, tumor stage, and development of metastasis.^11^, ^12^

Genome‐wide assessment using somatic SCNAs provides useful information for identifying overall genomic profiles of cancer cells.^11^, ^12^ The accumulation of SCNAs contributes to tumor heterogeneity and consequently striking differences in the presence of SCNAs between primary and metastatic sites.^13^ The accumulation of SCNAs plays important roles in tumor progression in RCC as well as other types of cancer.^14^

Here, we examined the clinicopathological impact of SCNAs in a Japanese cohort using an array‐based comprehensive genomic methodology. The differences in molecular profiles and clinical outcome were carefully analyzed in the present study. Our aim was to identify the association of SCNAs present in ccRCC with clinicopathological findings. In addition, we attempted to show the genomic heterogeneity that occurred in ccRCC and the clinical impact of SCNA patterns.

## 2 MATERIALS AND METHODS

### 2.1 Patients

A total of 59 patients undergoing renal mass excision for ccRCC between January 2011 and June 2017 were enrolled in this study at Iwate Medical University. The 59 patients we examined were divided into two categories, including a first cohort (30 cases) and then a second cohort (29 cases) to validate the results of the first cohort. The fresh tissues were frozen in liquid nitrogen immediately after dissection. All tissue samples were confirmed to be ccRCC type based on their pathology and they were diagnosed according to the “WHO guidelines for tumors of the urinary system and male genital organs” with a slight modification.^15^, ^16^ The clinicopathological findings included sex, age, tumor size, tumor location, Fuhrman grade, necrosis, venous invasion, TNM stage, and the presence of metachronous metastasis as indicated by the Japanese Classification for Renal Cell Carcinoma (Table 1).^17^ The median duration of follow‐up of metachronous metastasis was 49 months (range, 3‐82 months). During this follow‐up period, 11 patients with metachronous metastasis died. In the present study, no patients with additional treatment, such as chemotherapy and radiotherapy, were included. Protocols were approved by the ethics committees and institutional review boards of participating centers (HG2018‐519).

**Table 1:** Clinicopathologic findings of clear cell renal cell carcinomas in patients in each cohort

Findings	1st cohort	2nd cohort
	Cases (%)	Cases (%)
Total	30	29
Sex		
Men	20 (66.7)	18 (62.1)
Women	10 (33.3)	11 (37.9)
Age, y, range (median)	32‐80 (68)	44‐82 (65)
Size, mm, range (median)	18‐85 (41.5)	15‐125 (48)
Locus		
Right	16 (53.3)	16 (55.2)
Left	14 (46.7)	13 (44.8)
Fuhrman grade		
Grade 2	22 (73.3)	14 (48.3)
Grade 3	8 (26.7)	15 (51.7)
Necrosis		
Present	6 (20.0)	12 (41.4)
Absent	24 (80.0)	17 (58.6)
Venous invasion		
Positive	2 (6.7)	9 (31.0)
Negative	28 (93.3)	20 (69.0)
pT stage		
pT1	24 (80.0)	17 (58.6)
pT2	3 (10.0)	5 (17.2)
pT3	3 (10.0)	7 (24.2)
Metachronous metastasis		
Positive	11 (36.7)	14 (48.3)
Negative	19 (63.3)	15 (51.7)
Survival		
Dead	7 (23.3)	4 (13.8)
Alive	23 (76.7)	25 (86.2)

### 2.2 DNA extraction

DNA was extracted from isolated normal and tumor tissues by sodium dodecyl sulfate lysis and proteinase K digestion, followed by a phenol‐chloroform procedure as reported previously.^18^

### 2.3 Estimation of tumor DNA content

Pathologic examination of hematoxylin and eosin stained tissue directly adjacent to the area used for single nucleotide polymorphism (SNP) array was performed to ensure that the region of tumor examined was as phenotypically homogenous as possible, to maximize tumor percentage in the tissue sampled and to minimize the presence of stroma and normal tissue. In addition, necrotic tissue was avoided.

The area of the selected tumor tissue that was adjacent to the sample site was quantitatively analyzed using digital pathology with Aperio Software (Leica Biosystems). Tissue sections were scanned on an Aperio AT2 scanner with an average scan time of 120 seconds (compression quality, 70). Images were analyzed using color deconvolution and colocalization. Aperio Image Analysis software (for measurement of tumor tissue area) was used. The ratio was obtained by dividing the area of the whole tumor tissue that included interstitial tissue by the area of tumor tissue without such interstitial tissue. The ratio was generally greater than 0.8 (80%‐90%). A representative figure is shown in Figure S1.

### 2.4 SNP array analysis

The Cytoscan HD (Affymetrix, UK) platform was used in all experiments. This array contains more than 1.9 million nonpolymorphic markers and over 740 000 SNP markers with an average intragenic marker spacing of 880 bps and intergenic marker spacing of 1737 bps. These platforms consist of microarrays containing nonpolymorphic probes for copy number variations (CNVs) from coding and noncoding regions of the human genome as well as polymorphic SNP probes. All procedures were carried out as instructed by the manufacturer. The hybridized slides bearing DNA marked with biotin, were analyzed with a GeneChip Scanner 3000 7G (Affymetrix) and the Chromosome Analysis Suite Software (Affymetrix). Definition of abnormalities required a minimum of (a) 50 consecutively duplicated probes, (b) 50 consecutively deleted probes, or (c) segments of loss of heterozygosity (LOH) larger than 3 Mb. Smaller alterations involving cancer‐associated genes were also investigated. Common CNVs were considered constitutional/likely benign. Candidate genes mapped to large altered segments were defined based on disease‐associated Online Mendelian Inheritance in Man genes, previously related to RCC and/or other types of cancers. The detailed methodology was described previously.^19^

### 2.5 Classification of copy number alteration

In the present study, we classified SCNAs into five subtypes, including gain, LOH, copy neutral LOH (CN‐LOH), mosaic, and mixed types. Whereas LOH was considered a cross chromosomal change that results in loss of the entire gene and the surrounding region, gain was defined as a cross chromosomal change that was caused by a gain of the entire gene and the surrounding region. CN‐LOH was defined as an occurrence of LOH in the absence of the allelic loss (copy number ≥ 2). On the other hand, a mosaic pattern was defined as a mixture of normal and abnormal cells with SCNAs. Finally, a mixed pattern was a mixture of greater than two SCNA patterns within one locus, such as LOH and LOH mosaic, or gain and LOH or gain and gain mosaic.

### 2.6 Hierarchical analysis of the copy number alterations

We conducted hierarchical cluster analysis to group the samples according to their SCNA patterns. This approach maximized homogeneity for each group and assured the greatest differences between the groups. This was achieved with open‐access clustering software (Cluster 3.0 software; http://bonsai.hgc.jp/~mdehoon/software/cluster/software.htm). The clustering algorithm was set to centroid linkage clustering, which is the standard hierarchical clustering method used in biological studies.

### 2.7 Statistical analysis

Differences in the clinicopathological variables were analyzed using *χ* ^2^ tests in Stat Mate‐III (Atom, Tokyo, Japan). Variables included sex, tumor size (divided into those ≤40 and >40 mm), tumor location, Fuhrman grade, necrosis, venous invasion, TNM stage, overall survival, and the presence of metachronous metastasis among the subgroups. Differences in age and tumor size distributions among the groups were evaluated using the Kruskal‐Wallis *H* test in Stat Mate‐III. A *P* < .05 was accepted as significant. The differences in the SCNA pattern between each subgroup were assessed with a Fisher exact test with an adjusted Bonferroni correction.

We calculated disease‐free survival (without metachronous metastasis) of the patients based on the date of the surgery and the date of the last follow‐up or patient metachronous metastasis. In addition, the overall survival of the patients was analyzed. The Cox proportional hazards regression model was used for univariate and multivariate survival analyses. The level of significance was accepted at *P <* .05, and the confidence interval (CI) was determined at the 95% level. Statistical analyses were conducted with the JMP Pro 13.0 software package (SAS Institute, Inc, Cary, NC) for Windows.

## 3 RESULTS

### 3.1 SCNAs in ccRCCs in the first cohort

The median total number of chromosomal aberrations per patient was 113.5, with an average of 12.5 gains (range, 1‐198), 21 LOHs (range, 6‐64), and 18 copy‐neutral LOHs (range, 5‐98), 3 mosaic (0‐279), and 31 mixed (0‐162) types.

The regions of gain detected in more than 50% of cases were located at 14q32.33 in the ccRCCs. Additionally, regions of LOH (more than 50% of ccRCC cases) and that of mixed type were at 14q24.3 and 4q13.2, and 3p 24.2, respectively. No copy‐neutral LOHs or mosaic types showing more than 50% of cases were found.

#### 3.1.1 Hierarchical clustering based on SCNA patterns in ccRCCs in the first cohort

**Figure 1:**
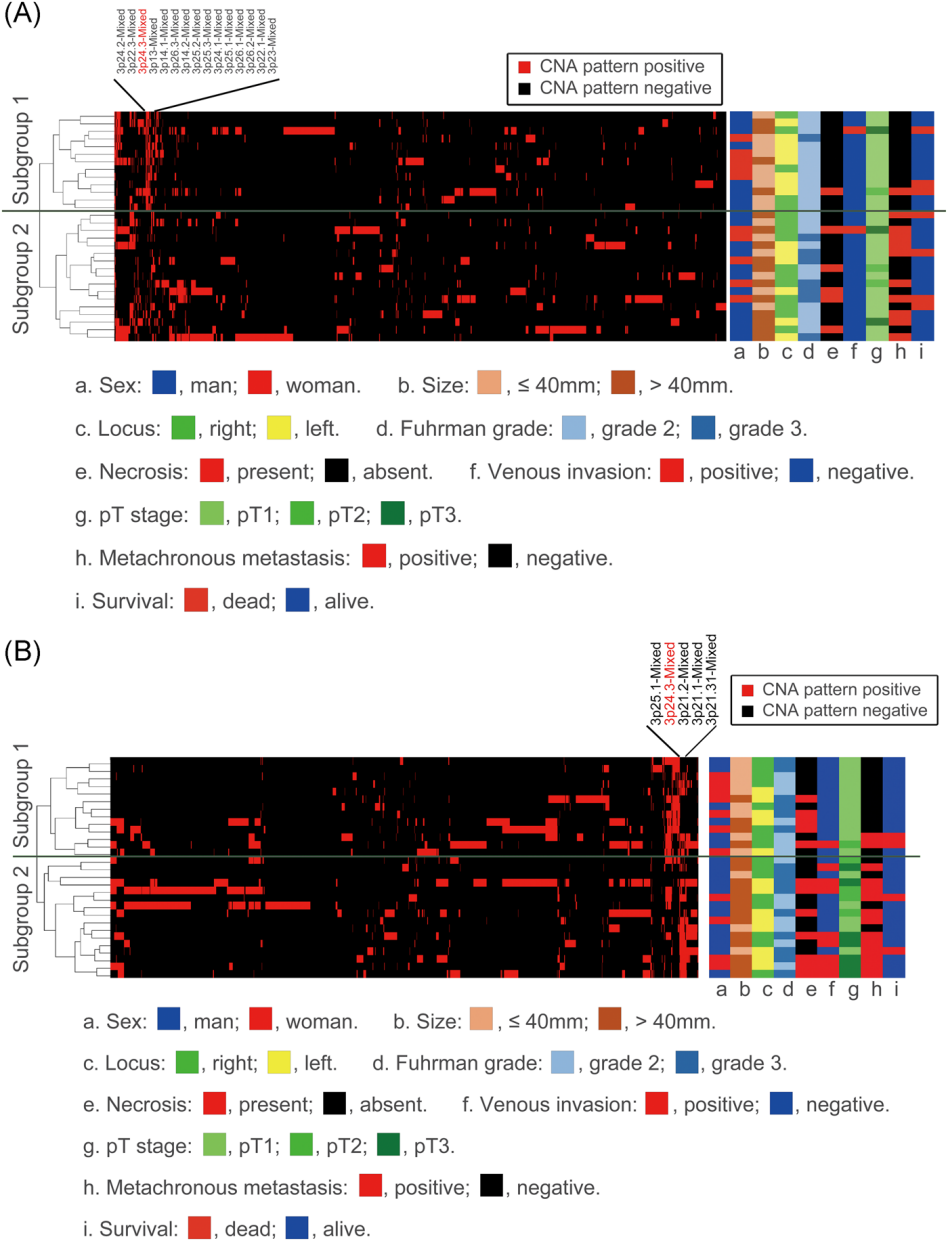
A, Hierarchical cluster analysis based on copy number alteration patterns in clear cell renal cell carcinoma in the first cohort. B, Hierarchical cluster analysis based on copy number alteration patterns in clear cell renal cell carcinoma in the second cohort [Color figure can be viewed at wileyonlinelibrary.com]

We assessed the SCNA pattern using hierarchical clustering. We identified two distinct subgroups (subgroup 1, 13 cases; subgroup 2, 17 cases) as shown in Figure 1A, in which the copy number alteration (CNA) marker in tumor tissue is indicated by the vertical line, and the horizontal lines denote “relatedness” between samples. Figure S2a was added to facilitate understanding.

#### 3.1.2 Differences in the clinicopathological findings between subgroups 1 and 2

No statistical differences in clinicopathological findings were found between the subgroups, including sex, age, tumor size, tumor location, Fuhrman grade, necrosis, venous invasion, TNM stage, and overall survival. However, we found that the frequency of metachronous metastasis was greater in subgroup 2 than in subgroup 1 (*P* = .0067; Table 2a).

**Table 2a:** Clinicopathologic findings of clear cell renal cell carcinomas based on each subgroup in the first cohort

	Subgroup 1 (%)	Subgroup 2 (%)	*P*
Total	13	17	
Sex			
Men	8 (61.5)	12 (70.6)	.7055
Women	5 (38.5)	5 (29.4)	
Age, y, range (median)	32‐80 (66)	49‐80 (68)	.6434
Size, mm, range (median)	22‐78 (35)	18‐85 (52)	.1394
Locus			
Right	5 (38.5)	11 (64.7)	.2685
Left	8 (61.5)	6 (35.3)	
Fuhrman grade			
Grade 2	12 (92.3)	10 (58.8)	.0924
Grade 3	1 (7.7)	7 (41.2)	
Necrosis			
Present	1 (7.7)	5 (29.4)	.1961
Absent	12 (92.3)	12 (70.6)	
Venous invasion			
Positive	1 (7.7)	1 (5.9)	1.0000
Negative	12 (92.3)	16 (94.1)	
pT stage			
pT1	11 (84.6)	13 (76.4)	.8584
pT2	1 (7.7)	2 (11.8)	
pT3	1 (7.7)	2 (11.8)	
Metachronous metastasis			
Positive	1 (7.7)	10 (58.8)	.0067
Negative	12 (92.3)	7 (41.2)	
Survival			
Dead	2 (15.4)	5 (29.4)	.4268
Alive	11 (84.6)	12 (70.6)	

**Table 2b:** Clinicopathologic findings of clear cell renal cell carcinomas based on each subgroup in the second cohort

	Subgroup 1 (%)	Subgroup 2 (%)	*P*
Total	13	16	
Sex			
Men	6 (46.2)	12(75.0)	.1426
Women	7 (53.8)	4(25.0)	
Age, y, range (median)	44‐79 (65)	50‐82(65)	.5985
Size, mm, range (median)	15‐72 (36)	21‐125(57.5)	.0283
Locus			
Right	8 (61.5)	8 (50.0)	.7107
Left	5 (38.5)	8 (50.0)	
Fuhrman grade			
Grade 2	7 (53.8)	7 (43.8)	.7152
Grade 3	6 (46.2)	9 (56.2)	
Necrosis			
Present	5 (38.5)	7 (43.8)	1.0000
Absent	8 (61.5)	9 (56.2)	
Venous invasion			
Positive	1 (7.7)	8 (50.0)	.0200
Negative	10 (90.9)	8 (50.0)	
pT stage			
pT1	12 (92.3)	5 (31.3)	.0025
pT2	1 (7.7)	4 (25.0)	
pT3	0 (0.0)	7 (43.7)	
Metachronous metastasis			
Positive	2 (15.4)	12 (75.0)	.0025
Negative	11 (84.6)	4 (25.0)	
Survival			
Dead	2 (15.4)	2 (12.5)	1.0000
Alive	11 (84.6)	14 (87.5)	

#### 3.1.3 Differences in the SCNA patterns between subgroups 1 and 2

**Figure 2:**
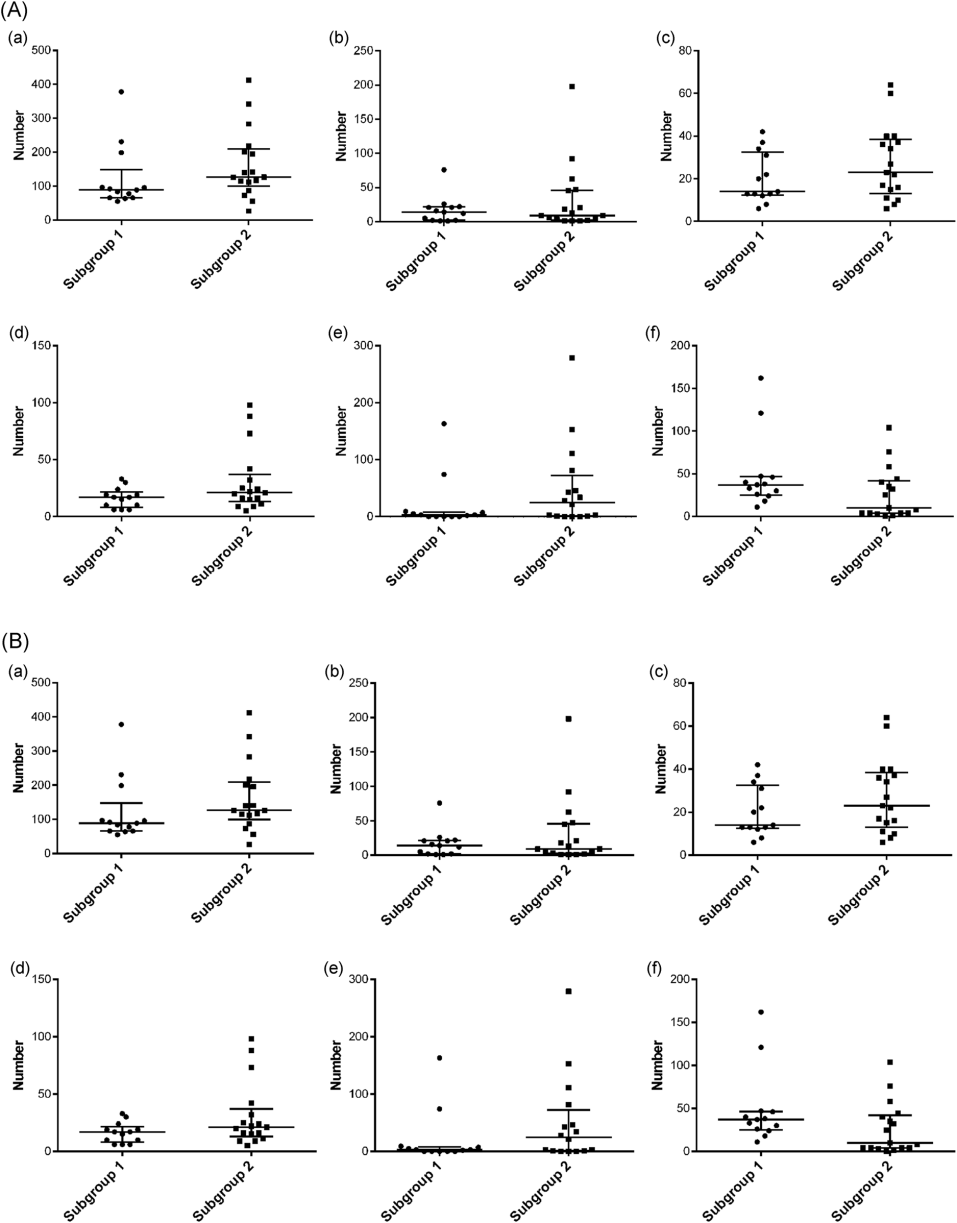
A, Number of loci with CNAs in subgroups 1 and 2 of cohort 2. (a) All CNA patterns, (b) gain pattern, (c) loss of heterozygosity pattern, (d) copy neutral loss of heterozygosity pattern, (e) mosaic pattern, (f) mixed pattern. B, Number of loci with CNAs in subgroups 1 and 2 of cohort 2. CNA, copy number alteration

Differences in the SCNAs between subgroups 1 and 2 are shown in Table 3. There were no significant differences between subgroups 1 and 2 in the total number of CNAs, median number of CN gains, LOH, CN‐LOH, and CN mosaic types. Although a difference was observed in the median number of CN mixed types between subgroups 1 and 2 (*P* = .0543), the difference fell short of statistical significance. The results are shown in Figure 2A.

**Table 3:** Somatic copy number alterations based on subgroups 1 and 2 in first and second cohorts

	1st cohort		2nd cohort	
	Subgroup 1	Subgroup 2	Subgroup 1	Subgroup 2
Median total number of chromosomal aberrations (range)	89 (1‐162)	127 (0‐279)	97 (0‐302)	156.5 (0‐541)
Median of gain (range)	14 (1‐76)	9 (1‐198)	21 (1‐121)	14 (0‐487)
Median of LOH (range)	14 (6‐42)	23 (6‐64)	14 (4‐67)	16 (0‐42)
Median of copy‐neutral LOH (range)	17 (6‐33)	21 (5‐198)	15 (3‐76)	15.5 (0‐33)
Median of mosaic type (range)	3 (0‐163)	21 (0‐279)	31 (0‐302)	44 (0‐541)
Median of mixed type (range)	37 (11‐162)	10 (0‐104)	23 (0‐59)	15 (3‐41)

Abbreviation: LOH, loss of heterozygosity.

Regions of gains detected in more than 50% of the cases were located at 5q21.2, 5q3, and 14q32.33 in subgroup 1; no gain was present in subgroup 2. LOHs detected in more than 50% of cases were found at 14q24.3‐LOH in subgroup 1 and 4q13.2‐LOH and 14q24.3‐LOH in subgroup 2. Although copy‐neutral LOH was not detected in subgroup 1, 3p21.31‐CNLOH was observed in subgroup 2. In addition, regions of mixed type detected in more than 50% of cases were harbored at 3p12.3‐mixed, 3p13‐mixed, 3p14.1‐mixed, 3p14.2‐mixed, 3p14.3‐mixed, 3p22.1‐mixed, 3p22.3‐mixed, 3p23‐mixed, 3p24.1‐mixed, 3p24.2‐mixed, 3p24.3‐mixed, 3p25.1‐mixed, 3p25.2‐mixed, 3p25.3‐mixed, 3p26.1‐mixed, 3p26.2‐mixed, and 3p26.3‐mixed in subgroup 1, but no mixed type was detected in subgroup 2. Finally, no mosaic type was found in any subgroup. We found that several CN mixed types were factors for differentiating each subgroup. The results are shown in Table 4a.

**Table 4a:** Significant differences in the frequencies of CNA patterns between subgroups 1 and 2 in clear cell renal cell carcinoma of the first cohort

CNA patterns	Subgroup 1 (%)	Subgroup 2 (%)	*P*
Total	13	17	
3p24.3‐mixed	13 (100.0)	1 (5.9)	.0002
3p24.1‐mixed	10 (76.9)	1 (5.9)	.1829
3p25.2‐mixed	10 (76.9)	1 (5.9)	.1829
3p26.3‐mixed	11 (84.6)	2 (11.8)	.2231
3p14.2‐mixed	9 (69.2)	1 (5.9)	.8377
3p13‐mixed	9 (69.2)	1 (5.9)	.8377
3p25.1‐mixed	9 (69.2)	1 (5.9)	.8377
3p14.1‐mixed	9 (69.2)	1 (5.9)	.8377

Abbreviation: CNA, copy number alteration.

**Table 4b:** Significant differences in the frequencies of CNA patterns between subgroups 1 and 2 in clear cell renal cell carcinoma of the second cohort

CNA patterns	Subgroup 1 (%)	Subgroup 2 (%)	*P*
Total	13	16	
3p24.3‐mixed	12 (92.3)	1 (0.0)	.0063
3p26.3‐mixed	9 (69.2)	0 (0.0)	.1460
3p26.2‐mixed	9 (69.2)	0 (0.0)	.1460

Abbreviation: CNA, copy number alteration.

#### 3.1.4 Disease (metachronous metastasis)‐free survival and clinicopathological findings in the stratified subgroups

**Figure 3:**
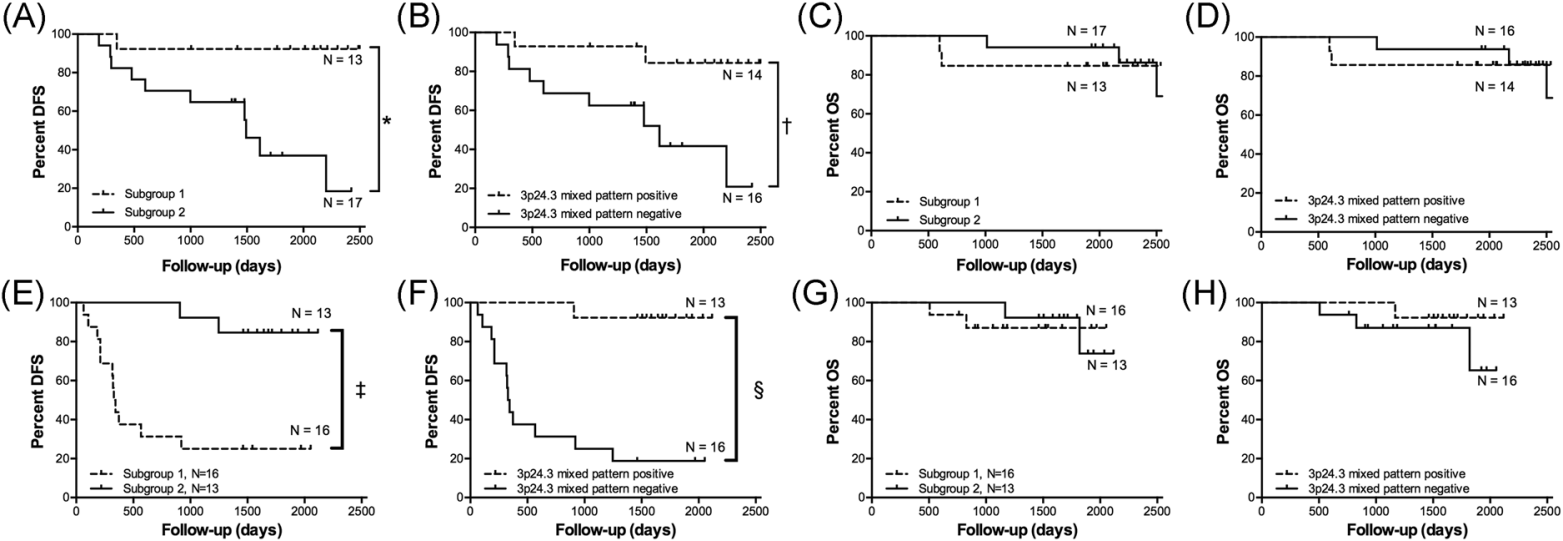
Kaplan‐Meier analyses. A, The rate of metachronous metastasis according to distinct subgroups based on copy number alteration in the first cohort. B, Association between the presence of metachronous metastasis and copy number alterations of mixed pattern at 3p24.3 in the first cohort. C, The rate of overall survival according to distinct subgroups based on copy number alteration in the first cohort. D, Association between overall survival and copy number alteration of mixed pattern at 3p24.3 in the first cohort. E, The rate of metachronous metastasis according to a distinct subgroup based on copy number alteration in the second cohort. F, Association between the presence of metachronous metastasis and copy number alteration of mixed pattern at 3p24.3 in the second cohort. G, The rate of overall survival according to a distinct subgroup based on copy number alterations in the second cohort. H, Association between the overall survival and copy number alterations of mixed pattern at 3p24.3 in the second cohort. *P = .0028, ^†^ *P* = .0087, ^‡^ *P* < .0001, ^§^ *P* = .0005. DFS, disease‐free survival; OS, overall survival

The proportion of metastasis‐free cases was 63.3% (19 of 30 ccRCCs). Kaplan‐Meier analysis was performed to determine and compare disease (metachronous metastasis)‐free survival and overall survival according to each stratified *SCNA pattern* (subgroups 1 and 2). Although there was no significant difference in the clinicopathological factors between subgroups 1 and 2, the presence of metachronous metastasis was correlated with subgroup 2 (Figure 3A). However, no correlation of each subgroup with overall survival could be found. Cox proportional hazards analysis was performed to determine and compare the disease‐free survival rates. We examined whether the clinicopathological findings and stratified subgroups were independent predictors of patient disease‐free survival. We used a univariate analysis for preliminary screening of the variables (Table 5a). This analysis was, in turn, followed by the application of a Cox proportional hazards model. The univariate analysis of patients with ccRCC (Table 5a) identified two factors (tumor size and stratified subgroup) associated with an increased frequency of metachronous metastasis.

**Table 5a:** Univariate and multivariate analyses of clinicopathologic findings and SCNA pattern subgroups as predictors of metachronous metastasis in clear cell renal cell carcinoma in the first cohort

Variables	Univariate analysis			Multivariate analysis		
	HR	95% CI	*P*	HR	95% CI	*P*
Size, mm						
>40 vs ≤40	5.125	1.316‐33.675	.0166*	2.752	0.668‐18.751	.2108
Fuhrman grade						
3 vs 2	3.421	0.981‐11.422	.0534			
Necrosis						
Present vs absent	2.132	0.465‐7.420	.2959			
Venous invasion						
Positive vs negative	1.332	0.072‐7.081	.7860			
pT stage			.5353			
pT2 vs pT1	1.901	0.278‐8.087	.4580			
pT3 vs pT1	2.282	0.338‐9.541	.3450			
pT3 vs pT2	1.200	0.143‐10.104	.8564			
CNA pattern subgroup						
2 vs 1	12.283	2.300‐227.169	.0015*	8.531	1.489‐162.522	.0482*

Abbreviations: CI, confidence interval; CNA, copy number alteration; HR, hazard ratio; SCNA, somatic copy number alteration.

* *P* < .05.

**Table 5b:** Univariate and multivariate analyses of clinicopathologic findings and SCNA pattern subgroups as predictors of metachronous metastasis in clear cell renal cell carcinoma of the second cohort

	Univariate analysis			Multivariate analysis		
Variables	HR	95% CI	*P*	HR	95% CI	*P*
Size, mm						
>40 vs ≤40	6.465	1.747‐41.722	.0035*	0.724	0.033‐7.746	.7909
Fuhrman grade						
3 vs 2	1.641	0.568‐5.002	.3579			
Necrosis						
Present vs absent	2.435	0.843‐7.426	.0990			
Venous invasion						
Positive vs negative	8.882	2.864‐30.892	.0002*	6.762	0.524‐87.231	.1354
pT stage			<.0001*			.3242
pT2 vs pT1	9.079	1.991‐46.288	.0056	3.658	0.344‐79.816	.2755
pT3 vs pT1	18.120	4.281‐102.586	<.0001*	1.257	0.053‐57.720	.8925
pT3 vs pT2	1.996	0.526‐9.332	.3181	0.344	0.044‐3.756	.3486
CNA pattern subgroup						
2 vs 1	9.256	2.470‐60.122	.0004*	9.274	1.259‐93.825	.0293*

Abbreviations: CI, confidence interval; CNA, copy number alteration; HR, hazard ratio; SCNA, somatic copy number alteration.

* *P* < .05.

Two factors were identified in the multivariate Cox proportional hazards analysis (Table 5a). Tumor subgroup classifications (subgroup 1 vs 2) remained significant predictors of disease‐free survival, even after controlling for the other variables. pT stage was not a factor associated with metachronous metastasis after adjusting for the effects of the other factors.

#### 3.1.5 Association of disease (metachronous metastasis)‐free survival and clinicopathological findings with a specific SCNA

We found that 3p24.3 mixed type was an independent factor for predicting good prognosis of ccRCC. Kaplan‐Meier analysis was performed to compare disease‐free survival according to 3p24.3 mixed type. The data showed that it was correlated with the presence of metachronous metastasis (Figure 3B). In univariate analysis, two factors, including tumor size and 3p24.3 mixed type, were correlated with metachronous metastasis (Table 6a). Of those, only 3p24.3 mixed type remained in multivariate analysis (Table 6a).

**Table 6a:** Univariate and multivariate analyses of clinicopathologic findings and CNA pattern (3p24.3‐mixed) as predictors of metachronous metastasis in clear cell renal cell carcinoma of the first cohort

	Univariate analysis			Multivariate analysis		
Variables	HR	95% CI	*P*	HR	95% CI	*P*
Size, mm						
>40 vs ≤40	5.125	1.316‐33.675	.0166*	3.443	0.841‐23.279	.0890
Fuhrman grade						
3 vs 2	3.421	0.981‐11.422	.0534			
Necrosis						
Present vs absent	2.132	0.465‐7.420	.2959			
Venous invasion						
Positive vs negative	1.332	0.072‐7.081	.7860			
pT stage			.5353			
pT2 vs pT1	1.901	0.278‐8.087	.4580			
pT3 vs pT1	2.282	0.338‐9.541	.3450			
pT3 vs pT2	1.200	0.143‐10.104	.8564			
CNA pattern (3p24.3‐mixed)						
Negative vs positive	6.241	1.576‐41.394	.0075*	4.432	1.079‐30.147	.0379*

Abbreviations: CI, confidence interval; CNA, copy number alteration; HR, hazard ratio.

* *P* < .05.

### 3.2 SCNAs in ccRCCs in the second cohort (the validation cohort)

The median total number of chromosomal aberrations per patient was 138, with averages of 23 gains (range, 23‐187), 15 LOHs (range, 2‐67), and 7 copy‐neutral LOHs (range, 2‐76), 3 mosaics (0‐541), and 20 mixed (0‐59) types.

The regions of gain detected in more than 50% of cases were located at 5q31.2, 5q31.3, and 14q32.33 in the ccRCCs. Additionally, regions of LOH (more than 50% of ccRCC cases) and that of mosaic type were at 14q24.3, 6q23.31, 19q13.42 and 3p12.3, 3p13, 3p14.1, respectively. No copy‐neutral LOHs or mixed types showing more than 50% of cases were found.

#### 3.2.1 Hierarchical clustering based on SCNA patterns in ccRCCs in the second cohort

Using hierarchical clustering, the SCNA pattern was also examined in the second cohort. Two distinct subgroups (subgroup 1, 13 cases; subgroup 2, 16 cases) were identified as shown in Figure 1B, in which the CNA marker in tumor tissue is indicated by the vertical line, and the horizontal lines denote “relatedness” between samples. Figure S2b facilitates understanding of the data.

#### 3.2.2 Differences in the clinicopathological findings between subgroups 1 and 2 in the second cohort

In the second cohort, some statistical differences in clinicopathological findings were found between the subgroups, including sex, age, tumor size, tumor location, Fuhrman grade, necrosis, venous invasion, and TNM stage. We found that tumor size, venous invasion, TNM stage, and the frequency of metachronous metastasis were greater in subgroup 2 than in subgroup 1 (tumor size, *P* = .0283; venous invasion, *P* = .0200; TNM stage, *P* = .0032; metachronous metastasis, *P* = .0025; Table 2b).

#### 3.2.3 Differences in the SCNA patterns between subgroups 1 and 2 in the second cohort

Differences in the SCNAs between subgroups 1 and 2 are shown in Table 3. Although a difference was observed in the median number of CN mixed type between subgroups 1 and 2 (*P* = .0978), the difference did not reach statistical significance. The results are shown in Figure 2B.

Regions of gains detected in more than 50% of the cases were located at 5q23.2, 5q23.3, 5q31.1, 5q31.2, 5q31.3, 5q32, 5q33.1, 5q33.2, 5q33.3, 5q34, 5q35.1, 5q35.2, and 5q35.3 in subgroup 1; no gain was present in subgroup 2. LOHs detected in more than 50% of the cases were found at 4q13.2‐LOH and 19q13.42‐LOH in subgroup 1 and 4q13.2‐LOH, 6q22.31‐LOH, and 10q26.13‐LOH in subgroup 2. However, copy‐neutral LOHs were not detected in more than 50% of the cases in each subgroup. In addition, no mosaic types were detected in subgroup 1, but mosaic types detected in more than 50% of cases were found at 3p12.3, 3p13, 3p14.1, 3p14.2, 3p14.3, 3p21.33, 3p22.1, 3p22.2, 3p22.3, and 3p23 in subgroup 2. Finally, regions of mixed type detected in more than 50% of the cases were harbored at 3p14.1‐mixed, 3p14.2‐mixed, 3p14.3‐mixed, 3p21.1‐mixed, 3p21.2‐mixed, 3p21.31‐mixed, 3p22.1‐mixed, 3p22.2‐mixed, 3p22.3‐mixed, 3p23‐mixed, 3p24.1‐mixed, 3p24.2‐mixed, 3p24.3‐mixed, 3p25.1‐mixed, 3p25.2‐mixed, 3p25.3‐mixed, 3p26.1‐mixed, 3p26.2‐mixed, and 3p26.3‐mixed in subgroup 1. In contrast, no mixed type was detected in subgroup 2. We found that several CN mixed types were factors for differentiating each subgroup. The results are shown in Table 4b.

#### 3.2.4 Disease (metachronous metastasis)‐free survival and clinicopathological findings in the stratified subgroups in the second cohort

The proportion of metastasis‐free cases in the second cohort was 51.7% (15 of 29 ccRCCs). Kaplan‐Meier analysis was performed to determine and compare disease (metachronous metastasis)‐free survival and overall survival according to each stratified SCNA pattern (subgroups 1 and 2). Although there was no significant difference in the clinicopathological factors and overall survival between subgroups 1 and 2, the presence of metachronous metastasis was correlated with subgroup 2 (Figure 3). Cox proportional hazards analysis was performed to determine and compare the disease‐free survival rates. We asked whether the clinicopathological findings and stratified subgroups were independent predictors of patient disease‐free survival. We used a univariate analysis for preliminary screening of the variables (Table 5b). This analysis was in turn followed by application of a Cox proportional hazards model. The univariate analysis of patients with ccRCC (Table 5b) identified four factors (tumor size, venous invasion, TNM stage, and stratified subgroup) that were associated with an increased frequency of metachronous metastasis.

Two factors were identified in the multivariate Cox proportional hazards analysis (Table 5b). Tumor subgroup classifications (subgroup 1 vs 2) remained significant predictors of disease‐free survival, even after controlling for the other variables. pT stage was not a factor associated with metachronous metastasis after adjusting for the effects of the other factors.

#### 3.2.5 Association of disease (metachronous metastasis)‐free survival, overall survival and clinicopathological findings with a specific SCNA in the second cohort

Kaplan‐Meier analysis was performed to compare disease‐free survival and overall survival with the 3p24.3 SCNA mixed type (Figure 3F and 3H). As a result, we found that although the 3p24.3 SCNA mixed type was not associated with overall survival, it was an independent factor to predict favorable prognosis of ccRCC. In univariate analysis, four factors, including tumor size, venous invasion, pT stage, and 3p24.3 mixed type were correlated with metachronous metastasis (Table 6b). Among those, only 3p24.3 mixed type remained after multivariate analysis (Table 6b).

**Table 6b:** Univariate and multivariate analyses of clinicopathologic findings and CNA pattern (3p24.3‐mixed) as predictors of metachronous metastasis in clear cell renal cell carcinoma of the second cohort

	Univariate analysis			Multivariate analysis		
Variables	HR	95% CI	*P*	HR	95% CI	*P*
Size, mm						
>40 vs ≤40	6.4649	1.747‐41.722	.0035*	1.510	0.142‐32.772	.7333
Fuhrman grade						
3 vs 2	1.641	0.568‐5.002	.3579			
Necrosis						
Present vs absent	2.435	0.843‐7.426	.0990			
Venous invasion						
Positive vs negative	8.882	2.864‐30.892	.0002*	5.652	0.511‐68.968	.1500
pT stage			<.0001*			.0521
pT2 vs pT1	9.079	1.991‐46.288	.0056*	20.951	1.612‐545.064	.0213
pT3 vs pT1	18.120	4.281‐102.586	<.0001*	50.840	2.013‐2696.288	.0167
pT3 vs pT2	1.996	0.526‐9.332	.3181	2.427	0.469‐20.833	.3127
CNA pattern (3p24.3‐mixed)						
Negative vs positive	20.324	3.972‐371.356	<.0001*	18.897	2.412‐411.608	.0043*

Abbreviations: CI, confidence interval; CNA, copy number alteration; HR, hazard ratio.

* *P* < .05.

## 4 DISCUSSION

It is well known that ccRCC is characterized by the presence of several SCNAs, as are other cancers, including gastric,^20^ colorectal,^21^ and ovarian cancers.^22^ Data show that multiple SCNAs may enhance tumor invasion and metastasis. ccRCC, however, can invade and/or metastasize irrespective of the low frequency of SCNAs, suggesting that the accumulation of SCNAs might be a minor facilitator of cancer invasion/metastasis in a subset of cancers.^23^ This hypothesis may be supported by the finding that the accumulation of SCNAs is not necessarily correlated with cancer invasion/metastasis in endometrial cancer, which is characterized by a low frequency of SCNAs.^23^ There are two distinct molecular subtypes, including microsatellite stable (MSS) and microsatellite instability (MSI) in cancer cells.^24^ In addition, MSS can be classified into chromosomal instability (CIN) that is characterized by multiple SCNAs and non‐CIN that is not accompanied by multiple SCNAs.^21^ Other mechanisms, such as DNA methylation and abnormal expression of microRNAs, other than multiple SCNAs, may be required for tumor progression in ccRCC.^21^, ^25^ Based on this theory, ccRCC may be assigned into the non‐CIN type. We suggest that the role of SCNAs in invasion/metastasis may vary with the type of cancer. Molecular profiles of tumor cells help to evaluate the carcinogenic course of ccRCC.^12^ Although we could classify the ccRCCs we examined into two subgroups (subgroups 1 and 2) in terms of SCNA pattern, there was no significant difference in the SCNA subtypes, including gain, LOH, CN‐LOH, mosaic, and mixed types between subgroups 1 and 2. However, we found that a 3p CN mixed type (LOH + LOH mosaic) was a significant factor for differentiating the subgroups in our patient cohort.

Next, we examined whether there was an independent prognostic factor among the 3p CN mixed type in ccRCC that we examined. Interestingly, we showed that 3p24.3 mixed type was an independent factor that predicted an excellent disease‐free prognosis in ccRCC. Although it is well known that a specific SCNA can be a prognostic factor to predict poor patient prognosis in various cancers including gastric,^26^ colorectal,^27^ and renal cell cancers,^28^ a prediction of good prognosis in ccRCC has not been reported as far as we know. Such association was supported by the finding that the 3p24.3 mixed type was correlated with disease‐free survival in the validation cohort (second cohort). Although we do not know why 3p24.3 mixed type (LOH + LOH mosaic) contributes to an excellent outcome in ccRCC, it is possible that the loss of oncogenic action located at 3p24.3 may be associated with a good prognosis for ccRCC (LOH and LOH mosaic pattern may cause loss of function).

We attempted to investigate the association of the 3p24.3 mixed type with overall survival in ccRCC. Unfortunately, no such association could be obtained in the present study. Thus, despite the finding that the 3p24.3 mixed type is correlated with disease‐free survival in ccRCC, the reason that there was no association of 3p24.3 mixed type with overall survival remains unknown. Further studies will be needed to identify the association.

We examined candidate oncogenes located at 3p24.3, given that loss of oncogenic function due to LOH and LOH mosaic pattern may contributes to a good prognosis of ccRCC. There are many candidate oncogenes located at 3p24.3, including *SGO1*, raftlin, lipid raft linker 1 (*RFTN1*), *ZNF385D*, high mobility group box protein 1 (*HMGB1*), *VENTX*, ribosomal protein L24 (*RPL24*), *RPL31, RANP7*, and phosducin‐like protein 3 (*PDCL3*). These were estimated to be highly expressed in various cancers in the Human Protein Atlas (https://www.proteinatlas.org).^29^ If low expression of those oncogenes occurred in SCNA of mixed type (LOH and LOH mosaic) in ccRCC, good prognosis of ccRCC may be expected. Among nine candidate oncogenes, high expression of four genes including *RFTN1, HMGB1, RPL24*, and *RPL31* were reported to be correlated with a poorer prognosis in cancers (conversely, one may say that low expression of such genes shows good prognosis). However, no correlation of the SCNA with expression of a specific gene showing the SCNA was found.

One report showed that high expression of *RFTN1* was closely associated with poor prognosis of ccRCC, compared with low expression (The Human Genome Atlas).^29^ Previous study has shown that *RFTN1* controls toll‐like receptor adaptor molecule 1 (*TICAM‐1*) signaling that regulates protein‐protein interactions between the toll‐like receptors and signal‐transduction components.^30^ Based on this finding, *RFTN1* may play a role via *TICAM*‐1 in renal carcinogenesis. Second, *HMGB1* is abundantly expressed in almost all human cells, and plays an important role as a chromatin‐associated nuclear protein.^31^ Overexpression of HMGB1 occurs in a variety of human cancers, such as prostate cancer,^32^ RCC,^33^ hepatocellular carcinoma,^34^ lung cancer,^35^ colorectal cancer,^36^ and gastric cancer,^37^ which may suggest a potential oncogenic role of *HMGB1*. This finding may mean that low expression of *HMGB1* is correlated with good prognosis in various cancers. Third, a previous study provided new evidence that *RPL24* expression is common in human breast tumors, but that depletion or structural alteration of *RPL24* can significantly impair human breast cancer cell viability.^38^ According to this finding, it is possible that low expression of *RPL24* could be correlated with good prognosis of cancers such as thyroid and renal cell cancers (The Human Genome Atlas).^29^ In addition, *RPL31* is a component of the large subunit of eukaryotic ribosomes. *RPL31* knockdown could mediate extraribosomal functions and regulate the function of tumor suppressor p53.^39^ Although this finding is not direct evidence for an association of RPL31 expression with cancer prognosis, low expression of *RPL31* may influence patients’ prognosis in cancer. In a previous study, high expression of *RPL31* was closely associated with poor prognosis in renal cell cancer (The Human Genome Atlas),^29^ suggesting inversely low expression of *RPL31* may be correlated with good prognosis of ccRCC. Finally, *PDCL3* is an interesting molecule in that *PDCL3* expression is regulated by hypoxia and plays an important role in the stability of VEGFR‐2. This finding may suggest that angiogenesis is a part of the hypoxia‐sensing mechanism that maintains physiological angiogenesis.^40^ The association of PDCL3 with cancer progression remains unknown, and further study will be needed. To summarize, we hypothesize that the loss or low expression of the above oncogenic genes may be caused by SCNA‐mixed type with LOH and LOH mosaic.

There are several limitations to this study. First, the number of patients enrolled in the study was small, particularly when compared to comprehensive “big data” analyses, such as those in TCGA.^12^ In addition, SCNA types that were used in the present study are different from those of previous comprehensive analyses.^12^, ^41^, ^42^ However, a correlation of a specific SCNA with patient prognosis, including disease‐free survival, was not observed in a “big data” cohort study (comparison of the present study with other big data studies, including TCGA^41^ and “Integrated Molecular Analysis of Clear‐cell Renal Cell Carcinoma,”^42^ as summarized in Supporting Information Table). In the present study, irrespective of small studies, we showed that the 3p24.3 mixed type is correlated with disease‐free survival, a finding that is supported by the validation cohort in which disease‐free survival was correlated with a favorable prognosis. Second, in retrospective cohort studies, a second cohort for validation purposes, in addition to the first cohort, may be necessary to identify the outcomes of patients with ccRCC. The present study, however, was limited to a single cohort. In the near future, we will attempt to validate the results presented here. Third, in the present study, we could not examine genetic differences in SCNA patterns between primary ccRCC and metastatic cancer tissues. Such information could be valuable; however, it is difficult to obtain frozen tumor tissues derived from metastatic lesions. In addition, it might be informative to compare the cancerous tissues with nearby normal tissues to determine whether the SCNA genotypes were strongly associated with tumor development. Finally, we could not examine the mutation status of whole genomes, such as whole exome sequence, in the present study. Further studies may be undertaken in the near future.

In conclusion, using cluster analysis of ccRCC, we examined the SCNA patterns including SCNA gain, LOH, CN‐LOH, mosaic, and mixed types. We found that they could be stratified into 2 distinct subgroups. Although there was no significant difference between subgroups 1 and 2 in the SCNAs we classified, a statistical difference in the mixed types (LOH and LOH mosaic) at 3p24.3 was found. The frequency of mixed type (LOH and LOH mosaic) at 3p24.3 in the metachronous metastasis was significantly higher in subgroup 1 than in subgroup 2. The integrated analysis of gene SCNAs pointed to several interesting genes as potential biomarkers for ccRCC although further studies need to be performed. Taken together, these results may be helpful in the understanding of renal carcinogenesis.

## CONFLICT OF INTERESTS

The authors declare that there are no conflict of interests.

## AUTHOR CONTRIBUTIONS

TT, who is the first author, constructed the figures and tables and performed statistical analyses. KI and MO performed histological diagnosis and statistical analysis. ES, RK, RT, and WO assisted with clinical data. TS, who is the corresponding author, contributed to the preparation of the manuscript and all aspects of data collection and analysis.

## ETHICS STATEMENT

This study was approved by the Ethical Research Committee, Iwate Medical University. All procedures were performed in accordance with the ethical standards of Iwate Medical University and with the Declaration of Helsinki. An alternative of informed consent (approved by the Institutional Review Board of Iwate Medical University) was obtained from all patients included in the study.

## Supporting information

Supplementary Figure 1. Histological findings of a representative case of clear cell renal cell carcinoma. The stromal component is surrounded by green lines. Total area of cancer tissue surrounded by the red square is 1,245,587.10 μm^2^, and the total amount of stromal areas are 64,335.90 μm^2^. Thus, the percentage of cancer cells is 94.8%. **(Author: this assumes that all cells are the same size?)**

Supplementary Figure 2. Heatmaps of each cohort based on 3p24.3‐mixed pattern. (A) First cohort. (B) Second cohort

Supporting information

## Data Availability

All data generated or analyzed during this study are included in this published article (and its supplementary information files).
