# Supplementary figures and images for: Comprehensive analysis of somatic copy number alterations in clear cell renal cell carcinoma
Source: Mol Carcinog. 2020 Feb 10;59(4):412–24. doi: 10.1002/mc.23164 (PMC7079091; doi:10.1002/mc.23164)

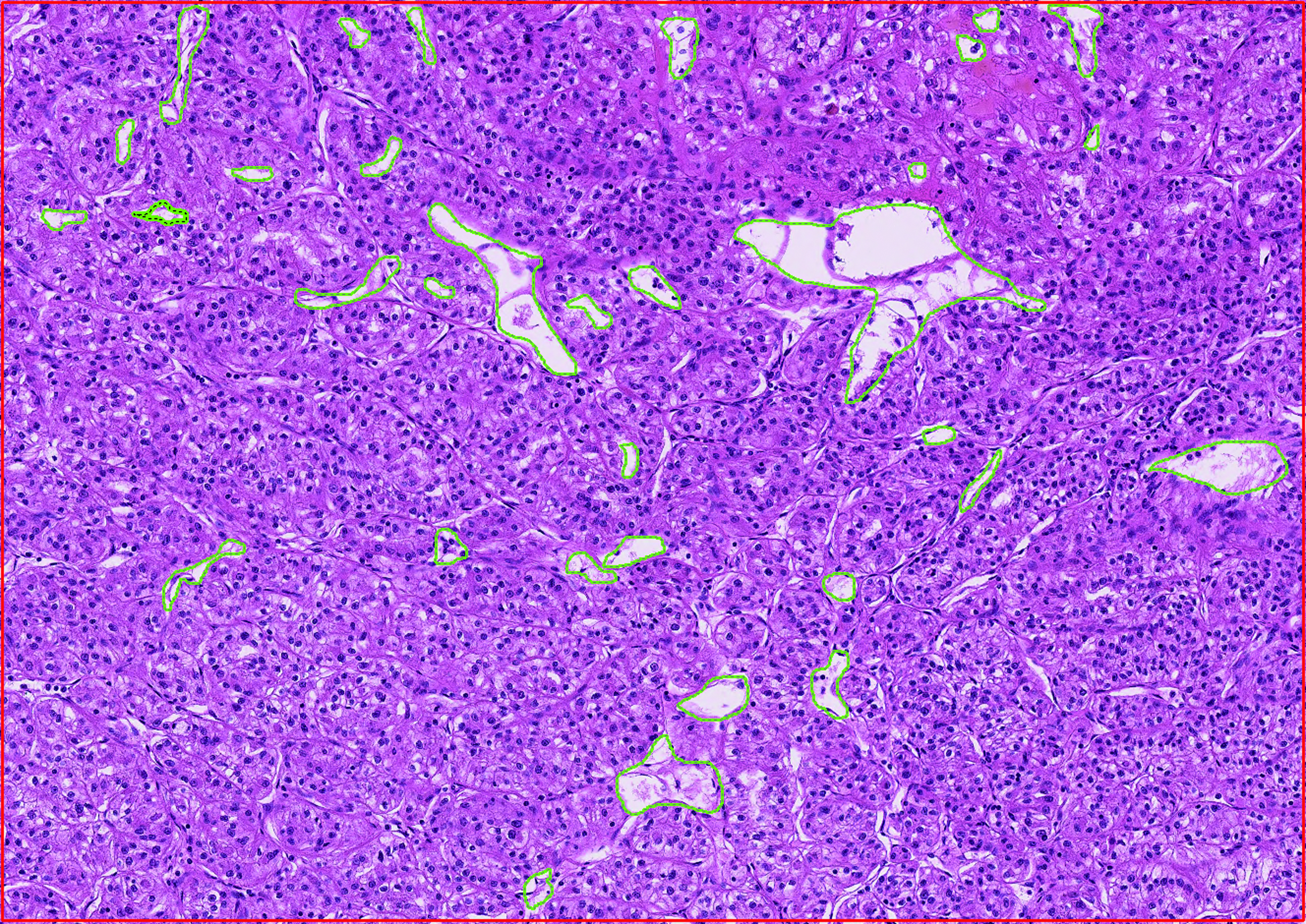

Supplement: Supplementary file 1 — Supplementary Figure 1. Histological findings of a representative case of clear cell renal cell carcinoma. The stromal component is surrounded by green lines. Total area of cancer tissue surrounded by the red square is 1,245,587.10 μm2, and the total amount of stromal areas are 64,335.90 μm2. Thus, the percentage of cancer cells is 94.8%. (Author: this assumes that all cells are the same size?) [file MC-59-412-s001.tif]

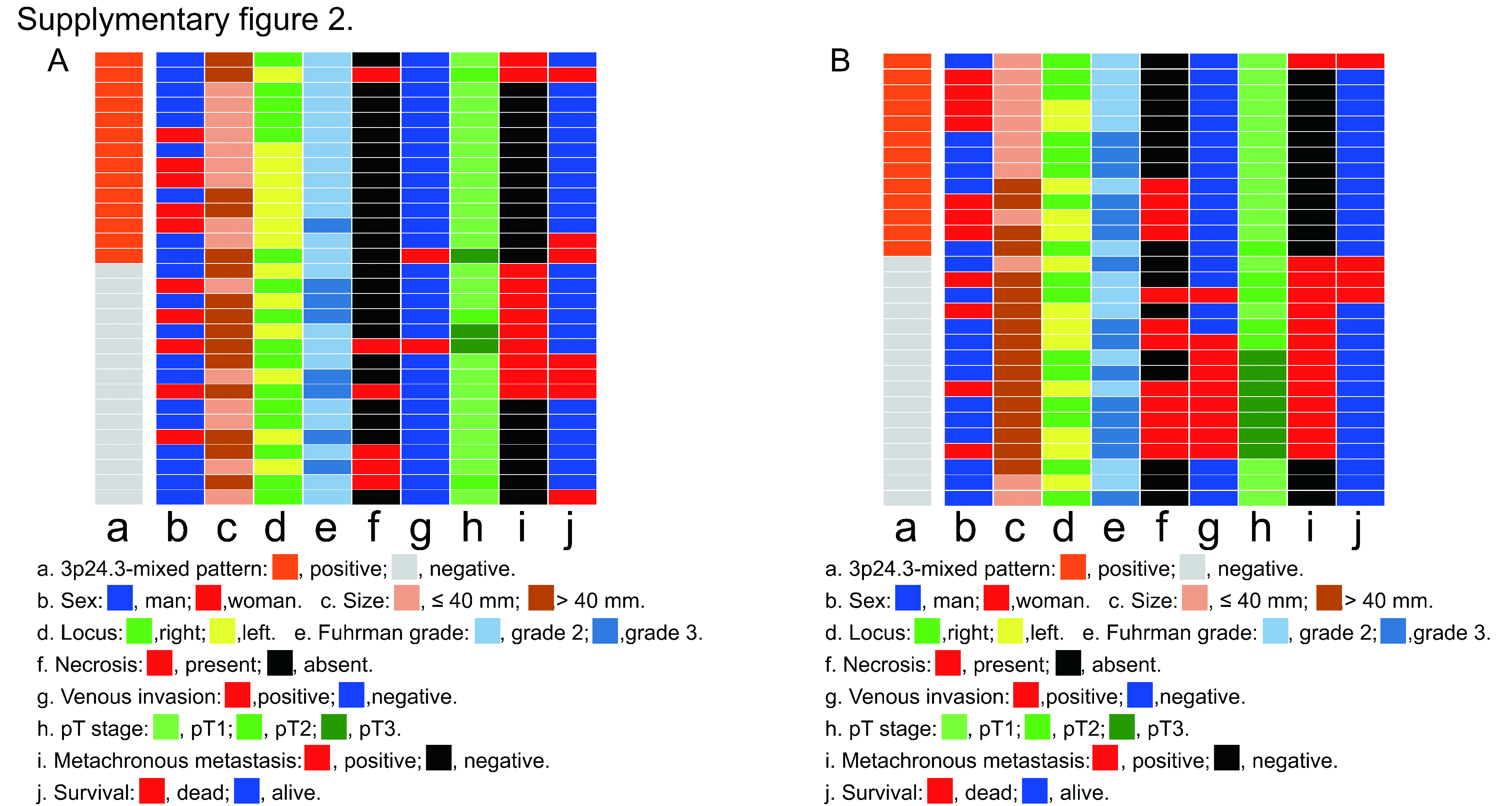

Supplement: Supplementary file 2 — Supplementary Figure 2. Heatmaps of each cohort based on 3p24.3‐mixed pattern. (A) First cohort. (B) Second cohort [file MC-59-412-s002.tif]
