# Supplementary material for: Comprehensive analysis of somatic copy number alterations in clear cell renal cell carcinoma
Source: Mol Carcinog. 2020 Feb 10;59(4):412–24. doi: 10.1002/mc.23164 (PMC7079091; doi:10.1002/mc.23164)
Supplement: Supplementary file 3 — Supporting information [file MC-59-412-s003.docx]

Supplementary Table. Comparison of the present study with other “big data” studies, including TCGA and “Integrated molecular analysis of clear-cell renal cell carcinoma”.

|  | This study | | Nature (TCGA) ^42)^ | Nature Genetics ^43)^ |
| --- | --- | --- | --- | --- |
| Platform | Affymetrix  CytoScan HD/750K Array | | Affymetrix  Genome-Wide Human SNP Array 6.0 | Affymetrix  GeneChip^®^ Human Mapping 250K Nsp Array |
| Probe number | 946,600 SNPs | | 906,600 SNPs | 262,000 SNPs |
| The evaluation of CNAs | Considered CNLOH and mosaic  Classification of each locus | | Unconsidered CNLOH and mosaic | Considered CNLOH  Unconsidered mosaic |
| Characteristic CNA | 3p24.3-mixed  (LOH and LOH mosaic) | | 3p11.2-LOH  3p12.1-3-LOH  3p14.1-2-LOH  3p21.1-LOH  3p26.1-LOH | 3p-LOH  5q-gain  9p-CNLOH  14q-CNLOH |
| Correlation of prognosis | 3p24.3-mixed positive cases are good prognosis | | Unconsidered prognosis | Unconsidered prognosis |
|  | Cases (%) | | Cases (%) | Cases (%) |
| Case number | 1st. cohort | 2nd. cohort |  |  |
|  | 30 | 29 | 446 | 228 |
| Fuhrman grade | G2　22 (73.3)  G3　8 (26.7) | G2　14 (48.3)  G3　15 (51.7) | G1　8 (1.8)  G2　188 (42.2)  G3　181 (40.6)  G4　68 (15.2) | G1　40 (17.5)  G2　138 (60.5)  G3　45 (19.7)  G4　5 (2.2) |
| Metachronous metastasis | 11 (36.7) | 14 (48.3) | 71 (15.9) | 50 (21.9) |
| T stage | T1　24 (80.0)  T2　3 (10.0)  T3　3 (10.0) | T1　17 (58.6)  T2　5 (17.2)  T3　7 (24.2) | T1　219 (49.1)  T2　52 (11.7)  T3　169 (37.9)  T4　6 (1.3) | T1　178 (78.1)  T2　18 (7.9)  T3　30 (13.2)  T4　2 (0.9) |
| Prognosis | OS  DFS | | OS  Unconsidered DFS | OS  Unconsidered DFS |
